# Differential Behavioral Responses of *Solenopsis invicta* (Hymenoptera: Formicidae) Workers Toward Nestmate and Non-Nestmate Corpses

**DOI:** 10.1093/jisesa/ieaa069

**Published:** 2020-07-29

**Authors:** Hua-Long Qiu, Chang-Sheng Qin, Eduardo G P Fox, De-Sen Wang, Yu-Rong He

**Affiliations:** 1 Guangdong Provincial Key Laboratory of Silviculture, Protection and Utilization/Guangdong Academy of Forestry, Guangzhou, Guangdong, China; 2 Instituto de Biofísica Carlos Chagas Filho, Centro de Ciências da Saúde, Universidade Federal do Rio de Janeiro, Rio de Janeiro, Brazil; 3 College of Agriculture, South China Agricultural University, Guangdong, Guangzhou, China

**Keywords:** myrmecology, invasive fire ants, necrophoresis, postmortem interval, evolution

## Abstract

The removal of corpses (aka ‘necrophoric behavior’) is critical to sanitation in ant colonies. However, little is known about differences in the necrophoric responses of *Solenopsis invicta* workers towards corpses of nestmates and non-nestmates. We introduced corpses of *S. invicta* workers from either intracolony (i.e., nestmate) or intercolony (i.e., non-nestmate) origin at the entrance of artificial nests, and recorded workers’ aggressive responses and necrophoric behaviors for analysis. *Solenopsis invicta* workers displayed distinct responses towards corpses of different origins. Specifically, resident workers were more likely to remove fresh non-nestmate corpses than nestmate corpses, but there was no difference regarding corpses that had been dead for 15 min or longer. Resident workers reacted more aggressively to, and removed more quickly, fresh non-nestmate corpses than corpses of their nestmates. On the other hand, there was no significant difference in the removal time between nestmate and non-nestmate corpses that had been dead for 15 min or longer. Resident workers always displayed stronger aggressiveness towards non-nestmate corpses than nestmate corpses, excepting to corpses that had been dead for 6 h, which elicited a response. No significant correlation between the removal times and aggressiveness levels were detected in any treatments. It remains to be tested whether this differential response is adaptive in how it influences colony fitness and competition.

The evolutionary and ecological success of social insects has been attributed to their sophisticated social behaviors, such as the reproductive division of labor between queens and workers, as well as task cooperation among worker castes (Hölldobler and Wilson 1990, Lach et al. 2010). However, there are drawbacks in group living. For example, the agglomeration inside humid enclosed nests facilitates the spread of pathogens, particularly from wastes and corpses (Hughes et al. 2002, Diez et al. 2012, Sun and Zhou 2013, Sun et al. 2018). As such, social insects have evolved complex corpse management behaviors that can reduce the risk of pathogens, ranging from prophylactic to therapeutic strategies. In the prophylactic strategies, old and injured workers will leave the nest to die outside, preventing the spread of potential pathogens in the nest (Heinze and Walter 2010). Prophylactic defensive behaviors act not only as a first barrier against the spread of pathogens within the colony, but also reduce the cost of nestmates investing in immunity activation (Schmid-Hempel 1998). In the therapeutic strategies, the corpses of nestmates are buried inside and/or removed from the nest by the resident workers (Renucci et al. 2010; Diez et al. 2011, 2013; Sun et al. 2013), and in rare cases, corpses may be cannibalized (Howard and Tschinkel 1976).

Corpse management behavior in social insects has attracted considerable attention, owing to its interesting characteristics and evolutionary importance. The removal of a corpse from the nest, also termed necrophoresis, is one of the best-described behaviors of social insects (Choe et al. 2009, Sun and Zhou 2013). A large number of ant species display necrophoric behavior on nestmates’ corpses within their colonies (Hölldobler and Wilson 1990). Most researches have focused on the necrophoric behavior of nestmates within a same colony (Haskins and Haskins 1974, Howard and Tschinkel 1976, Qiu et al. 2015). However, as a result of competition for food resources and territory among different colonies, fights among foragers and/or soldiers of competing colonies in close proximity may lead to accumulation of corpses (Hölldobler and Wilson 1990, Fadamiro et al. 2009, Sun et al. 2013). In such conditions, the encounters with corpses of non-nestmates within colonies can be relatively frequent in the field.

The red imported fire ant, *Solenopsis invicta* Buren, is not only a serious pest around the world, but is also a well-established model species for studies regarding ant behaviors such as grooming, social organization and necrophoresis (Tschinkel 2006; Gotzek and Ross 2009; Wang et al. 2013; Qiu et al. 2014, 2015, 2016). Regarding necrophoresis, previous reports have focused on the nestmate reactions to corpses of other nestmates or decoys (Sturgis and Gordon 2012). However, given the fact that *S. invicta* workers display different levels of aggression toward conspecific individuals with various degrees of genetic relationship (Meer and Alonso 2002), we hypothesized that *S. invicta* might show adaptive different necrophoric responses towards corpses of nestmates and non-nestmates. The results of this investigation will help us gain better understanding of the plasticity in social insects’ behavior when simultaneously dealing with interplaying chemical cues in nestmate and corpse recognition.

## Materials and Methods

The main hypothesis in this study is whether non-nestmate corpses would trigger aggressive behaviors by resident workers and affect the speed of removal of such corpses. This was investigated in different steps: 1) whether *S. invicta* workers would remove more non-nestmate corpses than nestmate corpses; 2) if the origin of removed corpses and their elapsed time since death have an effect on the necrophoric behavioral responses by resident workers; 3) whether there is a correlation between the speed of corpse removal and the displayed aggression level towards corpses of different origins. The details are described subsequently.

### Ant Colonies

Three colonies of *S. invicta* were collected from three different areas spread as least 5 km apart within the city of Guangzhou, Guangdong, P.R. China (one colony from inside the campus of South China University [E113.351977, N23.163690]), and two colonies from Zengcheng community (E113.727894, N23.175240; E113.619318, N23.117864) as to make sure that the ant colonies were physically independent. They were considered polygyne colonies, as several dealate queens were observed. In addition, after collection we observed the aggressiveness among workers from these different colonies to ensure their independence, by displaying stronger aggressive behavior toward workers of a different colony.

The collected colonies were conditioned into Teflon-painted plastic trays (45 × 40 × 20 cm), kept at 25 ± 1°C and 85 ± 3% relative humidity (RH), under a constant photoperiod of 12 h day^-1^. Colonies were fed larvae of *Tenebrio molitor* L. (Coleoptera: Tenebrionidae) purchased from the market and 25% sucrose in water every, every 2 d, ad libitum.

### Experimental Setup

**Fig. 1.**
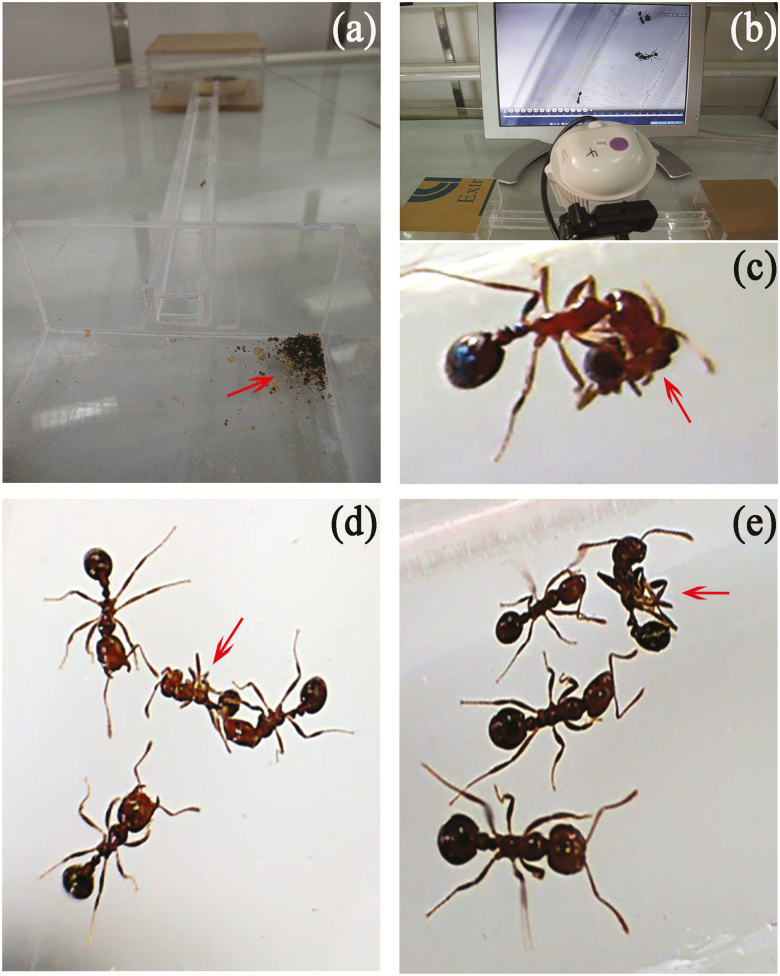
Experimental setup and displays of necrophoric behavior by *Solenopsis invicta* foragers. (a and b) A two-chamber inter-connected arena was used to assay the necrophoric behavior of *S. invicta*. One chamber was the foraging arena containing a refuse pile, while the other chamber held the nest core. These two chambers were interconnected by a corridor equipped with a Sony camcorder placed at about 10 cm, directly above. The behavior of workers in the corridor was recorded by the camcorder and compiled. (c) A worker removing a corpse. (d) Resident workers opening mandibules to attack a non-nestmate corpse. (e) Resident workers probing a nestmate corpse using antennae, displaying no aggression.

We established three standard laboratory subcolonies containing one queen and 200–300 workers, as well as 50 random broods picked out from each of the three colonies collected in the field. These subcolonies were kept inside a plaster nest (10 × 10 × 8 cm) held in an acrylic container connected to a foraging arena (10 × 8 × 8 cm) by an acrylic corridor (20 × 2 × 4 cm), wherein the behavioral responses of resident workers towards nestmate and non-nestmate corpses were observed. Subcolonies were acclimated under the aforementioned conditions for 2 wk before experimental procedures. Sony camcorders (FDR-AX60) were positioned directly on top of the interconnecting corridor at 10 cm from the nest entrance. The setup is illustrated in Fig. 1.

### Necrophoresis Towards Corpses

Nestmate and/or non-nestmate workers of similar size (media workers with head width roughly 1.15 mm) were haphazardly collected from each colony with fine tweezers and frozen to death at −20°C for 30 min. Their corpses were held in a constant temperature incubator at 25 ± 1°C, 85 ± 1% RH for 0, 15, 30, 60, or 360 min.

During each assay, one corpse was placed outside the nest at 10 cm from the entrance. Worker behavior, removal time (i.e., time from corpses presentation to being dropped in the refuse pile) and the total number of corpses discarded into refuse piles were videotaped and later compiled and calculated. Each test lasted 10 min, and repeated 21 times. If during the 10 min, the corpse was not removed, it was recorded as not removed but the removal time noted down as 10 min, thus the proportion of corpses removed was calculated based on the total number of 10-min observations.

The method for calculating the index of aggression was based on Diez et al. (2013):

$$\begin{array}{ll} Index\;of\;aggression\;= & \;[(N_{C}*0)+(N_{T}*1)+(N_{A}*2)+\\ & (N_{B}*3)]/(N_{C}+N_{T}+N_{A}+N_{B}), \end{array}$$

where *N*_C_ is the number of contacts (i.e., ant touching the corpse using antennae, with no aggressive display), *N*_T_ is the number of threats (i.e., weak aggression scored 1, where the ant approaches corpse with open mandibles), *N*_A_ is the number of attacks (i.e., medium aggression scored 2, where the ant moves back and forth rapidly around the corpse), *N*_B_ is the number of bites (i.e., strong aggression scored 3, where the ant bites the corpse, often presenting the gaster in stinging position).

In order to avoid possible short-term memory effects, we introduced intervals of least 30 min between each assay (Diez et al. 2011). We used a randomized block design with 10 treatments presenting either nestmate or non-nestmate corpses of the five different time intervals after death, employing three subcolonies with seven replicates for each treatment (i.e., totaling 210 experiments).

### Statistical Analyses

All data were analyzed with SPSS 20.0. Obtained data were tested for normality using the Shapiro–Wilk test. The numbers of nestmate and non-nestmate corpses removed by resident workers were compared using *t*-test. Aggression levels and removal times for corpses were analyzed using univariate GLM, with the origin of corpses and time post death as fixed explanatory variables, and the colony ID rated as a random factor. Wherever there was an interaction between the fixed variables, each factor was tested separately using one-way ANOVA or independent *t*-test in the fixed level of the counterpart factors. Pearson correlation method was used to investigate the correlation between corpses removal time and the level of aggression of resident workers.

## Results

**Fig. 2.**
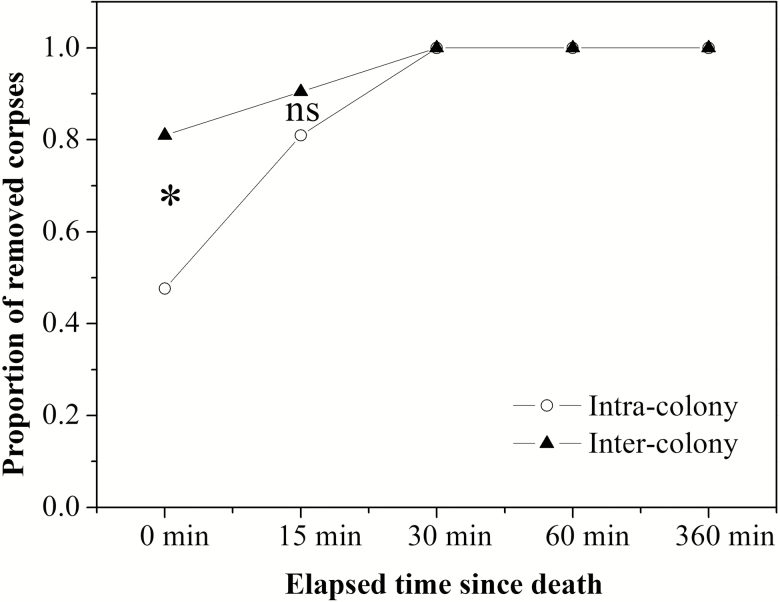
Proportion of removed ant corpses by resident *Solenopsis invicta* foragers as a function of time elapsed since death during the bioassay period. White circles and black triangles represent nestmate corpses (*n* = 21) and non-nestmate corpses (*n* = 21), respectively. An asterisk stands for significant difference; ‘ns’ meaning no significant difference between nestmates and non-nestmates (by *t*-test at *P* < 0.05).

The rates of fresh (i.e., 0 d) nestmate and non-nestmate corpses removed from the place of introduction were significantly different, with a higher proportion of non-nestmate corpses removed during the testing period (*t*-test, *t* = 5.08, *P* = 0.026; Fig. 2), but there was no difference between corpses that had been dead for 15 min (*t*-test, *t* = 1.54, *P* = 0.205; Fig. 2). Workers removed all corpses that had been dead for 30 min and beyond within the 10-min test period.

**Fig. 3.**
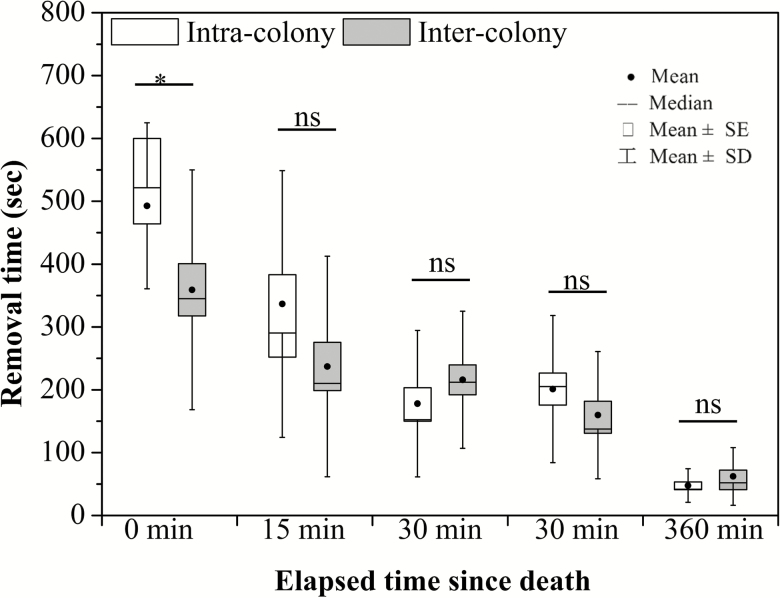
Times until corpse removal (mean ± SD) of nestmates or non-nestmates by resident *Solenopsis invicta* workers, grouped by different postmortem intervals. An asterisk stands for significant difference; ‘ns’ meaning no significant difference between nestmates and non-nestmates (by *t*-test at *P* < 0.05).

**Fig. 4.**
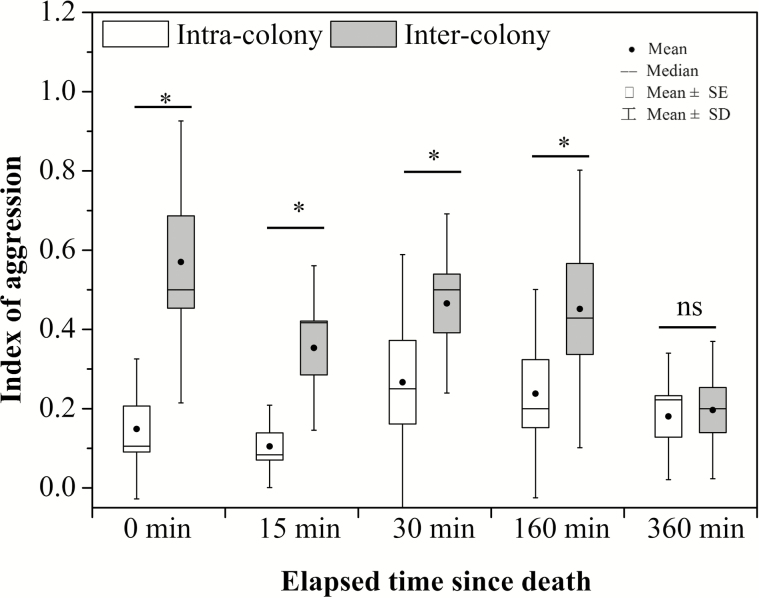
Calculated aggression indexes of resident *Solenopsis invicta* workers towards corpses of nestmates and non-nestmates, grouped by postmortem period. An asterisk stands for significant difference; ‘ns’ meaning no significant difference between nestmates and non-nestmates (by *t*-test at *P* < 0.05).

There was no significant difference between the tested colonies regarding the overall time taken to remove corpses (GLM: *F*_1, 210_ = 1.66, *P* = 0.20) nor between general aggression levels displayed by resident workers (GLM: *F*_1, 210_ = 0.06, *P* = 0.81). There was, however, a significant interaction between the origin of the corpses and their elapsed time post death (postmortem period) on the time taken for corpses to be removed (GLM: *F*_4, 210_ = 3.08, *P* = 0.017) and also on the displayed index of aggression by resident workers (GLM: *F*_4, 210_ = 3.55, *P* = 0.008). Therefore, we tested each factor separately through one-way ANOVA and independent *t*-test with the levels of the counterpart fixed factors. Specifically, towards nestmate corpses, resident workers took significantly different times to remove corpses with different postmortem periods (*F*_4, 100_ = 33.32, *P* < 0.001; Fig. 3), but no difference was observed in the aggression levels towards them (*F*_4, 100_ = 1.88, *P* = 0.119; Fig. 4). Regarding non-nestmate corpses with postmortem periods, resident workers not only took significantly different removal times to remove them (*F*_4, 100_ = 13.58, *P* < 0.001; Fig. 3), but also displayed significantly different aggressive behaviors towards them (*F*_4, 100_ = 5.54, *P* = 0.019; Fig. 4).

In order to clarify which postmortem periods triggered the most aggressive displays from resident workers, we analyzed the removal times and aggressive behaviors to nestmate and non-nestmate corpses grouped by same postmortem periods. Resident workers removed fresh (0-day) non-nestmate corpses more quickly than fresh nestmate corpses (Mann–Whitney *U* test, *P* = 0.011; Fig. 3), and were more aggressive towards fresh non-nestmate corpses when compared with fresh nestmate corpses (Mann–Whitney *U* test, *P* < 0.001; Fig. 4). For corpses dead for periods equal to or longer than 15 min there was no significant difference (15 min, Mann–Whitney *U* test, *P* = 0.109; 30 min, *t* = −1.092, *P* = 0.281; 60 min, *t* = 1.23, *P* = 0.226; 360 min, *t* = −1.25, *P* = 0.218; Fig. 3). However, resident workers still displayed significantly more aggressive behavior towards non-nestmate corpses of all postmortem periods—15 min (*t-*test, *t* = −4.94, *P* < 0.001), 30 min (*t-*test, *t* = −2.31, *P* < 0.001), and 60 min (*t-*test, *t* = −2.23, *P* = 0.032)—excepting 360 min (*t-*test, *t* = −0.334, *P* = 0.74) since death.

**Fig. 5.**
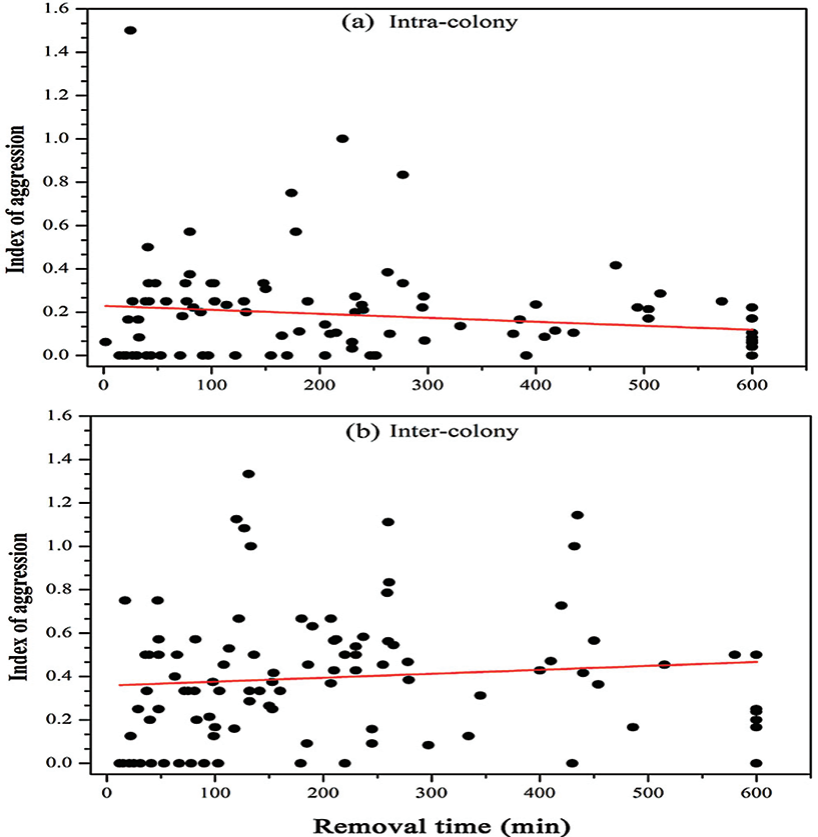
Pearson correlation between times to remove corpses and displayed aggressiveness indexes of resident *Solenopsis invicta* workers, towards corpses of nestmates (a) or non-nestmates (b).

There was no correlation between the removal times and aggressive levels towards corpses of nestmates (*r* = 0.006; Fig. 5a) nor non-nestmates (*r* = 0.017; Fig. 5b).

## Discussion

The main objective of this study was to investigate whether corpses from alien colonies would trigger different necrophoric behaviors by resident *S. invicta* workers. Obtained experimental results support the initial hypothesis that *S. invicta* workers would display different necrophoric behavior towards corpses of nestmates and non-nestmates, in the sense that non-nestmate corpses trigger stronger aggressive behavior get removed quicker than nestmate corpses.

The aggressive responses towards non-nestmate corpses may be partially due to the fact that they carry a significantly different cuticular chemistry signature compared with residents’. Cuticular chemicals profiles function as major cue in social insects ranging from nestmate and corpse recognition to queen attention and brood care (Martin and Drijfhout 2009, Sturgis and Gordon 2012, Richard and Hunt 2013). The difference would get recognized by the resident workers (Bos et al. 2012, van Wilgenburg et al. 2012) eliciting aggressive responses and quicker removal from the colony. In natural conditions, foragers of other colonies may occasionally approach the nest entrance and be met by competitor food foragers (Garnas et al. 2007). In such cases, resident workers would probably attack and perhaps kill such non-nestmate ants and intruders in a display which is akin to the observed, particularly with fresh dead bodies. Ant corpses may simultaneously emit chemical cuticular signals of nestmate-recognition and corpse-recognition cues. Therefore, it is possible that freshly freeze-killed corpses are perceived as living individuals on a first contact, and regarded as a potential threat by resident workers. It can be seen from observations that aggressive behaviors decrease with increasing postmortem periods down to a basal level displayed to nestmate corpses, particularly from 6 h postdeath, implying that either resident workers may no longer be able to discriminate non-nestmate from nestmate corpses (through loss of meaningful cuticular chemistry signature) or that the bodies are no longer perceived as potential threats for the colony.

‘Increased death cue’ and ‘diminished vital sign’ are two mainly reported strategies of corpse recognition involved in cuticular chemicals, and several studies have described accumulation or loss of chemical cues on the surface of ant corpses under necrophoric behavior (Sun and Zhou 2013, Qin et al. 2020). Mainly, the accumulation of oleic acid and, to a lesser degree, of caproic acid, are reported to elicit necrophoric behaviors, such as with the archaic ant *Myrmecia vindex* Smith, F. (Formicidae: Myrmeciinae) workers displaying a burying reaction or even transporting corpse-contaminated objects to refuse piles (Haskins and Haskins 1974). As examples of chemical changes following death, the compounds known as dolichodial and iridomyrmecin present in the pygidial glands of *Linepithema humile* Mayr (Formicidae: Dolichoderinae) workers and cuticle are reported to vanish from corpses by 40 min postmortem (Choe et al. 2009).

As a final note, it should be mentioned that corpse recognition in social insects may not always depend on cuticular chemicals, as there is some evidence suggesting that tactile cues could also be involved. For instance, in the termite *Reticulitermes virginicus*, both tactile and chemical stimuli are needed to elicit workers’ necrophoric response, while the loss of either cues would not lead to a necrophoric response (Ulyshen and Shelton 2012). Further chemical analyses and behavioral studies may thus examine these hypotheses with *S. invicta*.

In conclusion, our work reveals that the necrophoric behavior of *S. invicta* to nestmate and non-nestmate corpses is different. The broader question of how these behavioral variations can influence colony organization and fitness in the field is a matter pending further investigation.
